# Influence of Interaction Effects of the Different Pollenizers on the Blatina Variety (*Vitis vinifera* L.) Grape Cluster and Seed Characteristics

**DOI:** 10.3390/plants11030420

**Published:** 2022-02-03

**Authors:** Tatjana Jovanović-Cvetković, Vide Šutalo, Muhammed Kupe, Sezai Ercisli, Aleksandar Životić, Boris Pašalić

**Affiliations:** 1 Faculty of Agriculture, University of Banja Luka, 78000 Banja Luka, Bosnia and Herzegovina; boris.pasalic@agro.unibl.org; 2 Federal Administration for Inspection Affairs, 71000 Sarajevo, Bosnia and Herzegovina; videsutalo@hotmail.com; 3 Department of Horticulture, Faculty of Agriculture, Ataturk University, 25240 Erzurum, Turkey; muhammed.kupe@atauni.edu.tr (M.K.); sercisli@atauni.edu.tr (S.E.); 4 Faculty of Agriculture, Bijeljina University, 76300 Bijeljina, Bosnia and Herzegovina; aleksandar.zivotic@ubn.rs.ba

**Keywords:** grape, pollination, fertilization, grape quality, cluster, seed

## Abstract

Indigenous grape varieties represent a significant potential for viticultural diversification. Due to fertilization problems, certain varieties from this group require suitable pollenizers for successful fertilization and in order to achieve high-quality grapes. The study was conducted during the years 2016 and 2017 in the vineyard in Herzegovina (southern part of Bosnia and Herzegovina). The aim of this research is to define a suitable pollenizer for the ‘Blatina’ variety, which has a functionally female flower. Manual pollination was performed with five different pollenizers during the flowering period by applying pollen to the ‘Blatina’ variety inflorescence during the full bloom stage in the early morning hours. Pollinated inflorescences were isolated, marked, and monitored until the end of the vegetation, while open-pollinated clusters were the control group. The most important characteristics of grape clusters and seeds were analyzed. The best results during the research were obtained by open pollination. The significant effect of the pollenizers was registered in parameters: cluster mass, mass of grape berries in the cluster, number of grape berries per cluster, and average seed mass. Varieties ‘Žilavka’ and ‘Vranac’ had better characteristics compared to other pollenizer varieties. The results show that the Blatina variety production with a greater number of pollenizers ensures stable yields.

## 1. Introduction

The grapevine reproductive development has a direct effect on yield. It also impacts berry and wine quality by affecting traits such as berry and cluster size, cluster compactness, and berry skin to pulp ratio. A better understanding of reproductive processes such as gamete development, fertilization, seed development, and fruit-set is of great importance for yield and grape quality management [1].

Even though pollination is a widespread biological process in cultivated grapevines, there are still some uncertainties about the consequences of cross-fertilization regarding grape productivity and quality [2]. In the grapevine, the morphological development of inflorescences is well known; however, less information is available on the variety’s relationship regarding compatibility during cross-pollination and cross-breeding as well as the potential of different cross-breeds. Certain studies show that the grapevine has rather complicated fertilization biology [3]. This was a subject of several studies that studied pollination and fertilization in wild grapevine relatives and also some local, economically important varieties that have problems in the process of fertilization and fruit setting [4,5,6,7,8,9,10,11,12,13,14].

According to [3], several papers have cited that the characteristics of the microsporogenesis process are the main problem in the fertilization process, while in rare cases, one of the causes may be self-pollination incompatibility (self-incompatibility). The same author states that the effect of the pollenizer pollen on the maternal plant is classified into xenia (effects on the endosperm and embryo) and metaxenia (effects on the surrounding tissue–berry flesh). The analysis of [15] shows that it is not possible to make such a simple division, keeping in mind the interaction effect that pollenizer pollen can have on the maternal plant as well as the complexity of the analyzed effects. Some authors consider the influence of pollenizer pollen on the maternal plant through better reproductive abilities (viability and pollen germination) and more successful fertilization, while some papers have analyzed the specific influence of pollenizer pollen on changes in some berry and seed characteristics. The analysis of pollen characteristics, such as viability, germination capacity, and development characteristics of pollen tubes, is very important in defining varietal compositions for new vineyards and in breeding programs [1,3,16,17,18,19,20,21,22,23,24]. According to [25], pollen viability and pollen germination capacity are related to varieties, nutrition conditions, and environmental factors. The effect of the pollenizer pollen on certain fruit characteristics of maternal plants has been the subject of research in a number of fruit species, of which apples [26,27,28], pears [29], sour cherries [30], and cherries [31] have economic importance for our production conditions.

The grapevine cultivation in Bosnia and Herzegovina is traditionally associated with the production area of Herzegovina, i.e., the southern part of the country where the modified Mediterranean climate prevails. Grape and wine production have great importance for this region. In recent years, a lot of work has been done in terms of the introduction of indigenous varieties into production. According to literature statements [32], a significant number of indigenous grapevine varieties have been recorded in Herzegovina, among which there is a small number of commercially significant and, notably, more non-commercially significant, old, and less cultivated varieties. Only a small number of indigenous varieties are significantly used in production, and many of these varieties, which are well adapted to local environmental conditions, are on the verge of total extinction. Detailed research on the genetic diversity of old varieties [33] and their use and conservation potential is particularly important for the purpose of their sustainable use [34].

**Figure 1:**
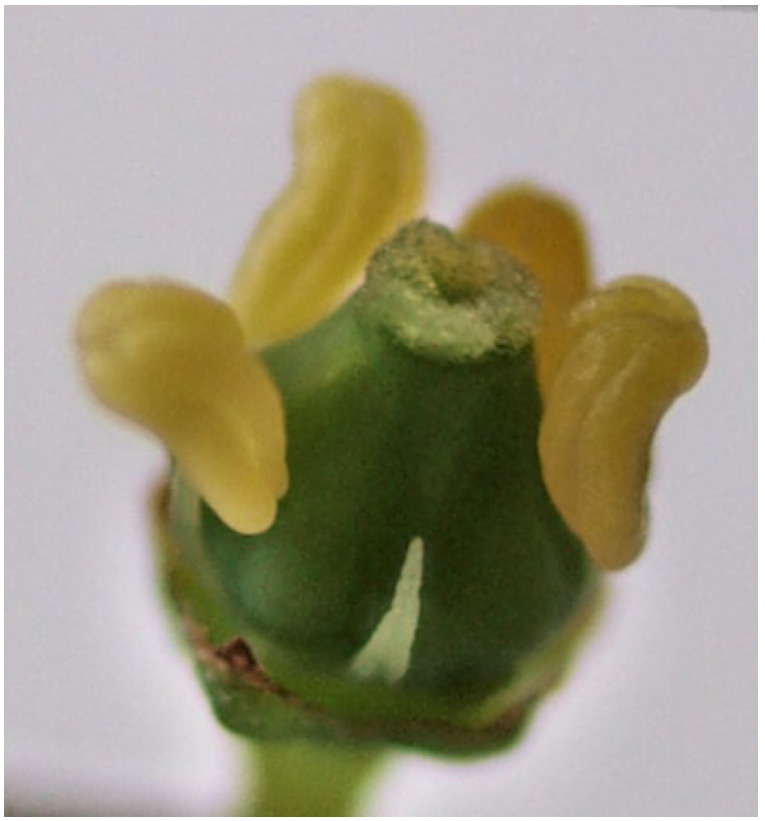
Morphological appearance of the flower of the ‘Blatina’ variety, just before the rejection of the cap of the crown leaves [41].

**Figure 2:**
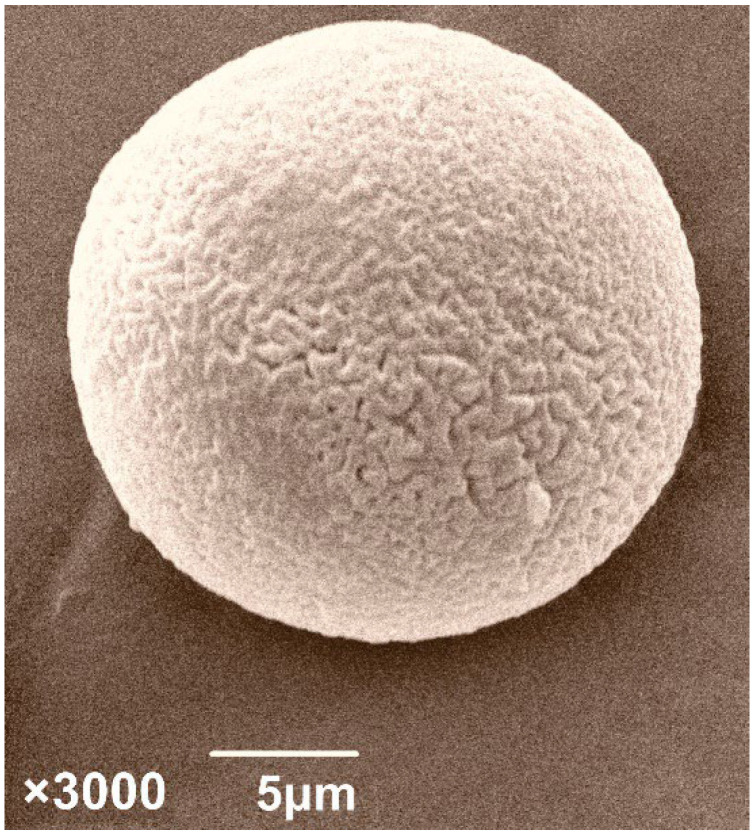
Hydrated pollen grain of the ‘Blatina’ variety without colpi and apertures that would enable pollen germination [40,41].

‘Blatina’ is an indigenous grapevine variety of Bosnia and Herzegovina used in red vine production [35]. It is present in the region of Herzegovina, which, due to its production importance, has been the subject of many studies, not only in Bosnia and Herzegovina but also in the surrounding countries [36,37,38]. ‘Blatina’ is a variety with a functionally female flower, and this has a great influence on the proper fruit setting and, thus, the yield (Figure 1). Although most cultivated grapevines have perfect flowers, with a normal ovary, pistil, and fully developed upright anthers, pollination insufficiency that results in millerandage (hens and chicks) is a worldwide problem that occurs in almost all grapevine varieties to varying degrees. Plants of one variety that have hermaphroditic and functionally female flowers can be found in regular production [39]. Certain research indicates that the occurrence of functionally female flowers is rare [24] in varieties with special significance for cultivation in certain regions. The pollen of the variety ‘Blatina’ is morphologically sterile [40] because it does not have fully formed openings for germination, so it does not have the ability to germinate, which is confirmed by the fact that in the conditions of self-fertilization, this variety lacks a fruit setting (Figure 2). Research on this variety in the previous period unequivocally indicates the fact that the reproductive characteristics of the variety ‘Blatina’ affect morphological sterility, and, for successful fertilization and fruiting, it is necessary to grow this variety with other varieties that can play the role of pollenizers. Finding varieties suitable for pollination of ‘Blatina’ is of great practical importance in raising new vineyards. Pollination and fertilization are the basic factors affecting fruit setting volume, and the most important goal of fruit producers is obtaining high quantity and quality yield in the horticultural industry, which depends on a sufficient fruit setting [25].

This paper reports the effects of different pollenizers as the pollen source on cluster and seed characteristics of ‘Blatina’ grapes. The results of cross-pollination were considered from viticultural and variety improvement viewpoints. The obtained results have practical importance in the assessment of the most suitable pollenizer for the Blatina variety based on the grape quality characteristics that are important in its regular, commercial production. Defining the most favorable pollenizer is of special importance not only for vine producers in Bosnia and Herzegovina but also for producers in the surrounding countries where this variety is grown.

## 2. Results

### 2.1. Pollen Germination

**Figure 3:**
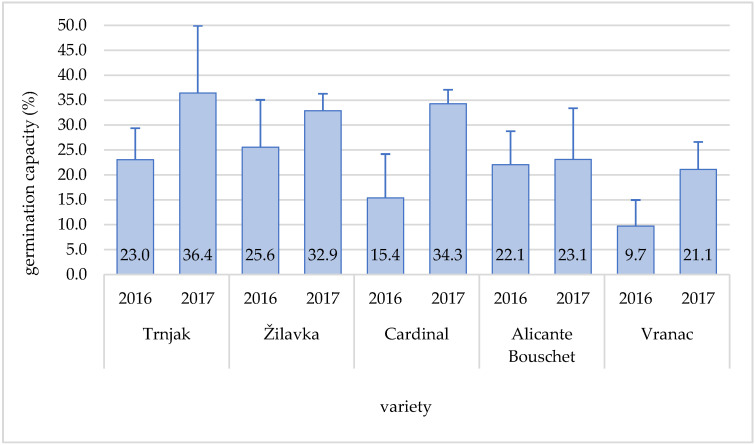
Pollen germination capacity of tested varieties during the research period (2016–2017).

Quality issue–grapevine pollen functional abilities are of great importance for viticultural practice, primarily for assortment selection since one-variety vineyards can be raised only with a self-fertile variety, which can provide pollination and fertilization with their own quality pollen [42,43]. The highest pollen germination capacity during the research period was in the varieties ‘Trnjak’ (36.4%), ‘Cardinal’ (34.3%), and ‘Žilavka’ (32.9%) in 2017, and it was higher in all examined varieties in 2017 than in 2016 (Figure 3).

The difference in the germination capacity during the years of research, from the aspect of the variety, was especially pronounced in the ‘Vranac’, ‘Cardinal’, and ‘Trnjak’ varieties. The ‘Žilavka’ variety did not have much difference in pollen germination during the research years, while the ‘Alicante Bouschet’ variety had almost uniform germination capacity in both years.

### 2.2. Cluster and Berry Characteristics

In order to determine the effects of the pollination of the ‘Blatina’ grape variety with different pollenizers (including open pollination) during the period of two years, a two-way variance analysis at multivariate and univariate levels was applied, the results of which are shown in Table 1 and Table 2. A two-way (6 × 2) Anova with pollenizers (Open pollination, ‘Trnjak’, Cardinal, ‘Alicante Bouschet’, ‘Žilavka’, and ‘Vranac’) and research year (2016 and 2017) as between-subjects factors revealed the main effects of pollenizer (F_(30;390)_ = 2.53, *p* = 0.000) and year of research (F_(6;97)_ = 5.64, *p* = 0.000). These main effects were qualified by an interaction between pollenizer and research year (F_(30;390)_ = 1.54, *p* = 0.037).

At the univariate level (Table 2), it can be noticed that the parameters: grape berry number per cluster (F (5;102) = 2.52, *p* = 0.034), grape berry mass (F (5;102) = 2.99, *p* = 0.015), and seed number per grape berry (F (5;102) = 3.17, *p* = 0.011) are accountable for the main effect of factor interaction.

**Figure 4:**
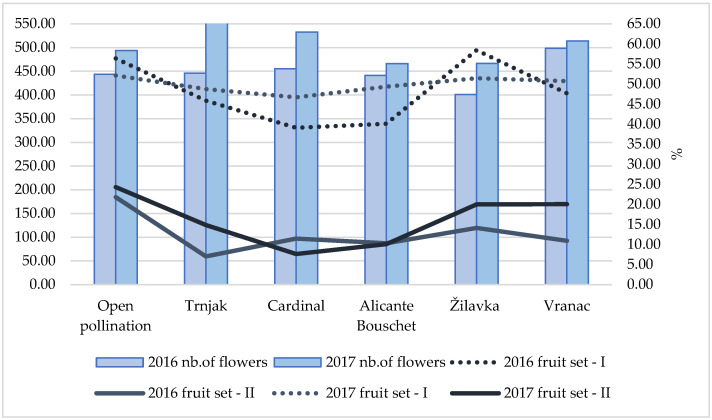
Fruit set (%) immediately after fertilization (fruit set I—initial fruit set immediately after fertilization) and in the harvest phenophase (fruit set II—final fruit set in the harvest phenophase).

**Figure 5:**
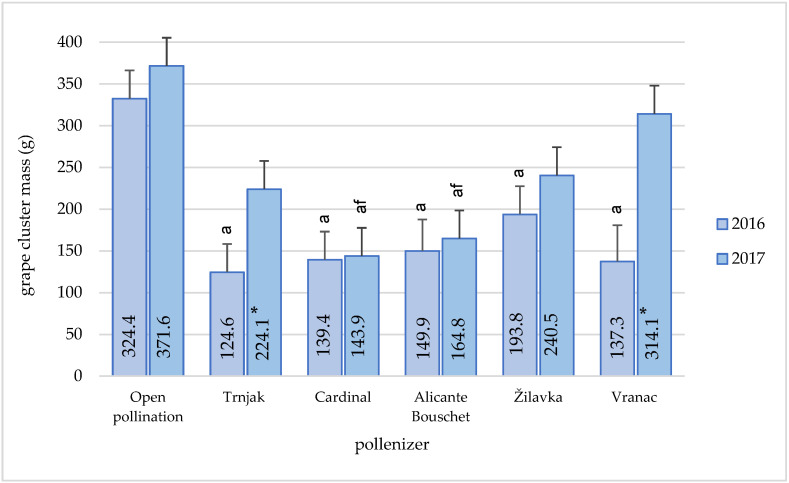
Pollenizer effect (‘Trnjak’, ‘Cardinal’, ‘Alicante Bouschet’, ‘Žilavka’, and ‘Vranac’) on Blatina variety cluster mass (g) during the research period (2016–2017). Means with different letters in a column (a—open pollination and f –‘Vranac’) are significantly different between pollenizers according to the ANOVA test (*p* ≤ 0.05); *—significant differences between years of research.

**Figure 6:**
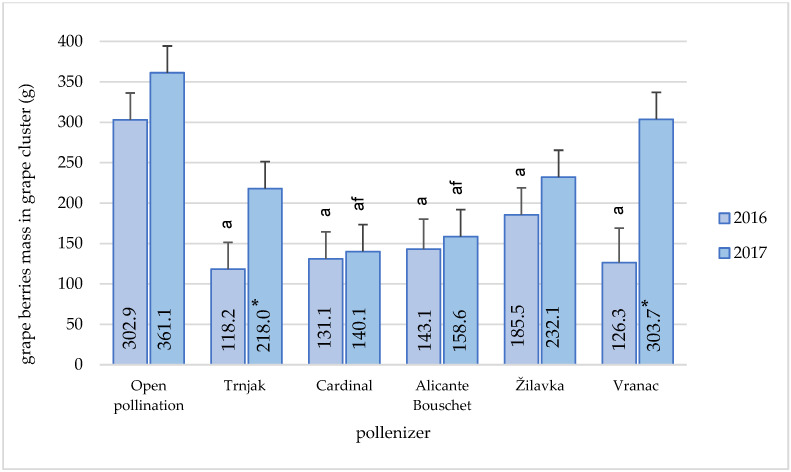
Pollenizer effect (‘Trnjak’, ‘Cardinal’, ‘Alicante Bouschet’, ‘Žilavka’, and ‘Vranac’) on Blatina variety grape berries mass in grape clusters (g) during the research period (2016–2017). Means with different letters in a column (a—open pollination and f—‘Vranac’) are significantly different between pollenizers according to the ANOVA test (*p* ≤ 0.05); *—significant differences between years of research.

**Figure 7:**
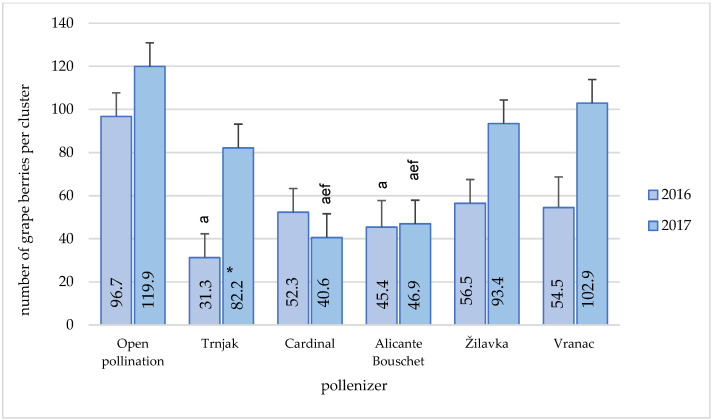
Pollenizer effect (‘Trnjak’, ‘Cardinal’, ‘Alicante Bouschet’, ‘Žilavka’, and ‘Vranac’) on Blatina variety number of berries per cluster during the research period (2016–2017). Means with different letters in a column (a—open pollination, e—‘Žilavka’ and f—‘Vranac’) are significantly different between pollenizers according to the ANOVA test (*p* ≤ 0.05); *—significant differences between years of research.

**Figure 8:**
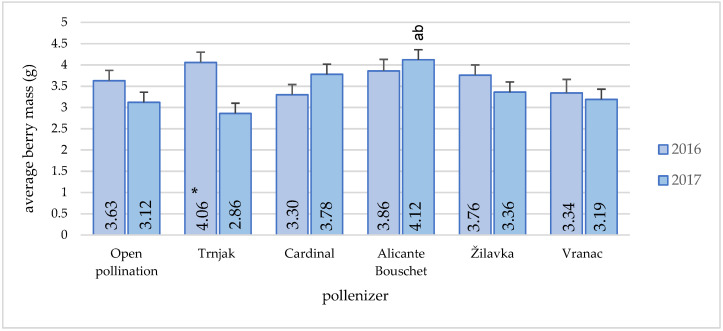
Pollenizer effect (‘Trnjak’, ‘Cardinal’, ‘Alicante Bouschet’, ‘Žilavka’, and ‘Vranac’) on the ‘Blatina’ variety average berry mass (g) during the research period (2016–2017). Means with different letters in a column (a—open pollination and b—‘Trnjak’are significantly different between pollenizers according to the ANOVA test (*p* ≤ 0.05); *—significant differences between years of research.

**Figure 9:**
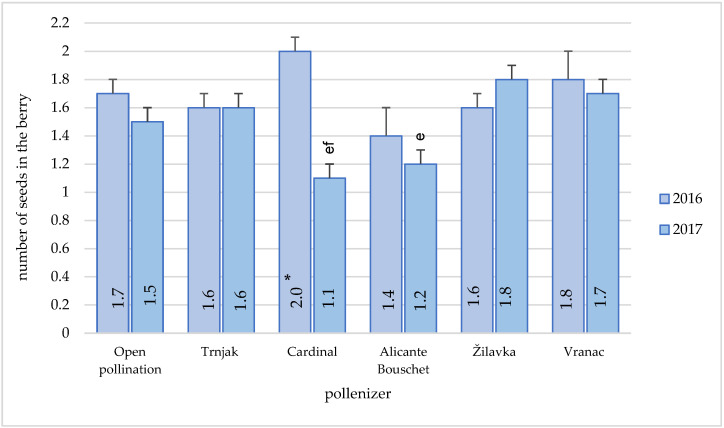
Pollenizer effect (‘Trnjak’, ‘Cardinal’, ‘Alicante Bouschet’, ‘Žilavka’, and ‘Vranac’) on the Blatina variety average number of seeds in the berry during the research period (2016-2017). Means with different letters in a column (e—‘Žilavka’ and f—‘Vranac’) are significantly different between pollenizers according to the ANOVA test (*p* ≤ 0.05); *—significant differences between years of research.

Analyzing the pollenizer as a factor, it can be stated that the parameters-cluster mass (F (5;102) = 10.13, *p* = 0.000), grape berries mass in the cluster (F (5;102) = 9.06, *p* = 0.000), and grape berry number per cluster (F (5;102)) = 9.14, *p* = 0.000)—are responsible for the high level of differences, while no statistically significant differences were found in the other parameters. Analyzing the year of research as a factor at the univariate level, the parameters cluster mass (F (1;102) = 9.92, *p* = 0.002), grape berries mass in the cluster (F (1;102) = 11.62, *p* = 0.001), and grape berry number per cluster (F (1;102) = 14.20, *p* = 0.000) are responsible for the differences as well as number of seeds per grape berry (F (1;102) = 6.10, *p* = 0.015). Post-hoc analysis using Tukey’s HSD test showed that the differences between the pollenizers within the ‘Blatina’ clusters contributed to the statistically significant difference only in cluster mass, mass of the grape berries in the cluster, and number of grape berries per cluster; these are labeled with different letters (a–f) in the columns in Figure 4, Figure 5, Figure 6, Figure 7, Figure 8 and Figure 9. In order to more clearly understand the influence of pollenizers on the Blatina variety grape and seed characteristics, an analysis of the fruit set percentage was conducted (Figure 4).

The initial fruit set during both years of the study was relatively uniform in all combinations and ranged from 40.06% (‘Alicante Bouschet’ in 2016) to 58.42% (‘Žilavka’ in 2016). In most combinations, this percentage was close to 50.0%. Slightly better fertilization was recorded during 2017, which could be related to the weather conditions during the fertilization phase. In the time of flowering and fertilization in 2017, slightly less precipitation and higher (more favorable) temperatures for fertilization were recorded compared to 2016, which was characterized by higher amounts of precipitation in that period. Final fruit set indicated a significant difference in pollinizer influence. The best final fruit set was registered in the conditions of open pollination (21.79%—in 2016 or 24.29%—in 2017). During 2017, a high percentage of final fruit set was registered with the combination of the varieties ‘Vranac’ (20.02%), ‘Žilavka’ (20.01%), and ‘Trnjak’ (14.86%), which, among other things, led to the larger size of berries and the higher number of berries per cluster.

The cluster mass is directly related to the grapevine yield. The pollen sources remarkably influenced the cluster mass [14]. The highest ‘Blatina’ variety cluster mass (Figure 5) in our study was obtained in the open pollination conditions (respectively, 332.4 g in 2016 and 371.6 g in 2017) during both research years. This was especially pronounced in 2017, when this difference, compared to all other pollinizers, was statistically significantly higher. ‘Vranac’ as a pollinizer influenced the formation of statistically significantly larger clusters compared to the results obtained with the varieties ‘Cardinal’ and ‘Alicante Bouschet’. No statistically significant differences were found in other mutual comparisons.

In 2017, in addition to open pollination, the ‘Vranac’ variety (314.1 g) had a great influence on the cluster mass, which caused a statistically significant higher cluster mass compared to the pollenizer varieties ‘Cardinal’ (143.9 g) and ‘Alicante Bouschet’ 164.8 g). Although a slightly higher amount of precipitation was recorded in 2016, it did not have a significant positive effect on the cluster mass as well as on other analyzed characteristics.

A similar effect of different pollenizers was found in the analysis of the berries mass in the cluster (Figure 6). In that case, during 2017, open pollination resulted in the highest mass of grape berries in the cluster.

During that year, the statistically significantly lower berries mass in the cluster was recorded in the variety ‘Cardinal’ (140.1 g) compared to open pollination (361.1 g) and cross-pollination with the ‘Vranac’ variety (303.7 g). The berries mass in the cluster in the open pollination conditions was 18.9% higher compared to pollination with the variety ‘Vranac’. In the conditions of open pollination, a statistically significant higher mass of berries in the cluster was recorded compared to other pollenizers, especially compared to the varieties ‘Alicante Bouschet’ and ‘Cardinal’ (127.68% and 157.74%). Other differences in the berries mass in the cluster during 2017 did not have statistical significance. In 2016, the berries mass in the cluster in open pollination conditions (302.9 g) was statistically significantly higher than the mass achieved by pollination with other pollenizers, where a relatively uniform effect on this grape cluster characteristic was recorded. Significant variations between the years of observation were found in the ‘Trnjak and ‘Vranac’ varieties. The berries mass in the cluster in the conditions of open pollination was higher by 63.29% compared to the berries mass obtained by this variety’s pollination. The difference in the berries mass obtained by open pollination was higher by 111.67% compared to pollination with the variety ‘Alicante Bouschet’ and by 156.26% compared to pollination with the variety ‘Trnjak’.

In 2016, the highest number of berries per cluster (Figure 7) was obtained by open pollination (96.7), and it was statistically significantly higher compared to the ‘Trnjak’ (31.3) and ‘Alicante Bouschet’ (45.4) varieties.

In 2017, the lowest number of berries per cluster was recorded when ‘Cardinal’ (40.6) and ‘Alicante Bouschet’ (46.9) were used as pollenizers. These results were significantly lower than the numbers achieved by open pollination (119.9) and pollination with the ‘Žilavka’ (93.4) and ‘Vranac’ varieties (102.9). The number of berries in the cluster was also significantly lower compared to the open pollination. The increase in the number of berries per cluster in open pollination conditions ranged from 16.52% for the variety ‘Vranac’ to 195.32% for the variety ‘Cardinal’. The stated difference for the variety ‘Cardinal’ can be partly explained by the specifics of this table variety. The difference between the research years was found only in the ‘Trnjak’ variety.

The influence of pollenizers on the average berry mass was not statistically significant (Figure 8) except for the ‘Alicante Bouschet’ variety, whose pollination achieved the highest average berry mass (4.12 g).

The average berry mass was statistically higher in open pollination conditions (3.12 g) compared to the conditions where Trnjak variety was used as a pollinizer (2.86 g). In this variety, a difference was found at the year level in terms of the average berry mass. However, from an agronomic aspect, it is evident that the number of berries per cluster had a significant effect of great importance on the berry mass, keeping in mind that all combinations with a larger number of berries per cluster had a lower average berry mass. The berry mass was in the values range stated by [13] for the ‘Blatina’ variety during the three-year research period, and it was 3.49 g.

Within our research, the berry mass in open pollination conditions was, on average, 9.04–12.08% higher compared to the varieties ‘Vranac’ and ‘Žilavka’, i.e., lower by 2.26–11.28% compared to the varieties ‘Cardinal’ and ‘Alicante Bouschet’.

### 2.3. Seed Development

The average number of seeds per grape berry of the ‘Blatina’ variety, during the research period, varied significantly with the ‘Cardinal’ variety as a pollenizer (Figure 9). In other combinations, this number was relatively even. In 2016, there were no significant differences between different pollenizers.

During 2017, the average number of seeds was statistically lower when the ‘Cardinal’ (1.1) and ‘Alicante Bouschet’ (1.2) varieties were used as pollenizers, compared to the Žilavka variety as a pollenizer. There were no statistically significant differences between the pollenizer varieties Cardinal and Vranac. Throughout the research period, there was no statistical difference between the pollenizers regarding this parameter.

**Figure 10:**
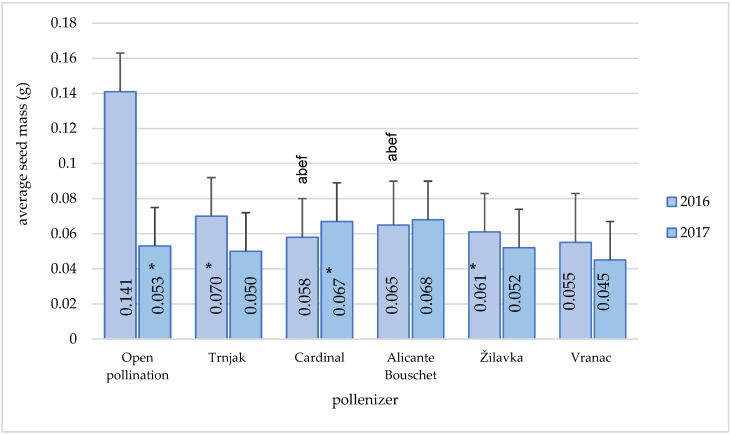
Pollenizer effect (‘Trnjak’, ‘Cardinal’, ‘Alicante Bouschet’, ‘Žilavka’, and ‘Vranac’) on the Blatina variety average seed mass (g) during the research period (2016–2017). Means with different letters in a column (a—open pollination, b—‘Trnjak’, e—‘Žilavka’ and f—‘Vranac’) are significantly different between pollenizers according to the ANOVA test (*p* ≤ 0.05); *—significant differences between years of research.

The average seed mass, depending on the pollenizer, ranged from 0.045 g (‘Vranac’) to 0.141 g (open pollination). Analysis of the average seed mass (Figure 10) shows the relatively uniform effects of different pollenizers and open pollination during 2017. Throughout the year of 2016, the average seed mass of the variety ‘Blatina’ in the conditions of pollination with the ‘Cardinal’ variety was statistically lower compared to open pollination and pollination with the ‘Trnjak’ and ‘Žilavka’ varieties. During the same year, the ‘Blatina’ variety average seed mass obtained by pollination with the ‘Alicante Bouschet’ variety was statistically lower compared to open pollination and pollination with the ‘Trnjak’ variety and statistically higher compared to pollination with the ‘Žilavka’ and ‘Vranac’ varieties. There was no statistical difference between the varieties ‘Cardinal’ and ‘Alicant Bouschet’ regarding average seed mass.

## 3. Discussion

### 3.1. Pollen Germination

The assessment of the functional ability of grapevine pollen is significantly conditioned by its germination capacity [23]. The ‘Vranac’ variety has shown very low pollen germination capacity, although it is often present in vineyards together with the ‘Blatina’ variety. During the research period, the ‘Žilavka’ variety showed the highest pollen germination capacity. According to [42], the high pollen germination capacity of the ‘Žilavka’ variety is one of the most represented in the Herzegovina region. Depending on the sucrose concentration in the solution (12.0% and 15.0%), it ranged from 31.5% and 29.0%. The high pollen germination capacity of the ‘Žilavka’ variety in the area of Herzegovina is also stated by [12]. According to them, the germination capacity of ‘Žilavka’ ranged from 18.21% to 42.52%, depending on the sucrose concentration in the solution and experimental year. There were slightly higher average values for the pollen germination of varieties ‘Alicante Bouschet’ and ‘Cardinal’, as stated by [21]. Based on another study’s results [25], a large number of varieties have high pollen germination capacity when 20.0% sucrose solution is used. The pollen germination of four grape varieties was examined by [3] in order to examine the occurrence of xenia and metaxenia. According to that study, pollen germination capacity ranged from 55.2% to 76.8%. According to research conducted by [20] during the analysis of Iranian varieties, the pollen germination capacity varied between 23.6% and 83.1%, whereby in most varieties, it was above 80.0% if germination was conducted immediately after sampling. According to [1], ‘Sangiovese’ pollen viability and germination capacity were, on average, 20.0% and 40.0%, respectively, and high viability and germination were also registered for ‘Corinthe Noir’, with an average value of 79.0% and 44.0% during the two seasons. In vitro pollen germination capacity that varies from 50.0% to 70.0% is considered normal, although many factors (genetic, physiological, environmental influence) can significantly influence that percentage [42].

### 3.2. Cluster and Berry Characteristics

Weather conditions during the study period had a significant impact on the degree of fertilization. This was especially pronounced on the initial germination in the varieties ‘Cardinal’ and ‘Alicante Bouschet’. Differences in final germination over the research period were smaller and somewhat more pronounced in the cultivars ‘Vranac’, ‘Žilavka’, and ‘Trnjak’. In the conditions of free fertilization during both years, the degree of the final embryo was over 20%. Similar data on fruit set are provided by [23] in research on the fertilization of the ‘Alphonse Lavallée’ variety. According to this study, the highest fruit set occurred when ‘Pembe Çekirdeksiz’ (20.2%) was used as a pollen source, while the lowest fruit set was recorded in self-fertilization (9.1%). The decision of whether fruits will be set generally depends on successful pollination, while further fruit growth is determined by fertilization, which initiates seed development [3,4].

The characteristics of ‘Blatina’ grapes in regular production often do not meet the producers’ expectations. The cluster usually does not have a satisfactory mass, which is directly reflected in the yield. The values obtained in this study indicate a large variation in the mass of the cluster (139.4–371.6 g), depending on the pollinator. The best results were achieved in the conditions of open pollination. The cultivar ‘Žilavka’ had a stable influence on the size of the grapes during the research period, as well as the cultivars ‘Vranac’ and ‘Trnjak’ during 2017. Slightly lower values of the ‘Blatina’ grape cluster average mass—228.28 g, during a three-year research period, are stated by [13]. We cannot exclude that the individual genotype plays a role in this phenomenon, which could be enhanced in certain varieties [1]. Open pollination increased the grape cluster mass by 55.6% and 62.1% when compared to varieties ‘Vranac’ and ‘Žilavka’ pollination. A significant increase was registered in comparison to the varieties ‘Trnjak’ (101.89%), ‘Alicante Bouschet’ (123.7%), and ‘Cardinal’ (148.5%). Similar data for the cluster mass of the ‘Blatina’ variety are given by [44]. They analyzed a large number of genotypes that were in the second stage of selection, created by fertilization of the ‘Blatina’ variety with the ‘Vranac’ and ‘Cardinal’ varieties and grafted on the Kober 5 BB rootstock. The cluster mass of the examined grapevine genotypes from the combination ‘Blatina’ × ‘Vranac’ ranged from 395.2 to 425.5 g. The genotypes that had the characteristics of the table grape varieties from the combination of cross-breeding ‘Blatina’ × ‘Cardinal’ had an average mass that ranged from 348.54 to 418.24g. In their study of the influence of different pollenizers, [14] also stated that the best results were achieved by open pollination. At the same time, [2] stated in their research that due to self-fertilization, the lowest results were achieved when it comes to cluster mass and that open pollination and manual pollination gave significantly better results. According to the same authors, this clearly indicates that the existence of an external pollen source can increase yield.

Under normal conditions, berry-setting percentages can be considered a varietal characteristic, but this is subject to various external and physiological factors [2], which was also stated in this research. The largest number of berries in the cluster within the research was recorded in the conditions of open pollination. Good results were also recorded with fertilization of the ‘Žilavka’ and ‘Vranac’ varieties. In open pollination conditions, the number of berries per cluster was high (96.7–119.9) and also significantly higher compared to the statements of [13]. They concluded that an average number of berries per cluster was 65.80 for the ‘Blatina’ variety in the vineyard, which indicates a significant variation of this characteristic depending on the production conditions and pollenizers. In his research, [3] also stated that the ‘Narince’ variety in open pollination conditions has a higher percentage of fruit-setting compared to self-fertilization. The pollenizers’ utilization in table grape varieties also affects significant differences, and it indicates a large variability in fruit setting percentage depending on the pollenizers [2].

Average berry weight was relatively uniform during the research period. A slightly larger mass of berries was found in the varieties ‘Alicante Bouschet’ and ‘Trnjak’, which is probably due to the smaller number of berries in the bunch. The obtained results are in accordance with the statements of other researchers. According to the data of [44], the berry mass of wine genotypes from the combination of the ‘Blatina’ × ‘Vranac’ varieties ranged from 3.20 to 3.40 g, while for table genotypes from the combination of the ‘Blatina’ × ‘Cardinal’ varieties’, grape berry mass ranged from 4.55 to 6.82 g. Based on results, Ref. [2] stated that the use of external pollen sources resulted in higher values regarding berry mass, length, and width in comparison to self-pollination. The berry mass in open pollination treatments was 26.6% and 22.8% higher than in the self-pollination treatment.

### 3.3. Seed Development

With the exception of the varieties ‘Alicante Bouschet’ and ‘Cardinal’, the number of seeds in the berry was quite uniform in other combinations and ranged from 1.5 to 1.8. According to [13], the average number of seeds per grape berry was 1.51 during the 2008–2010 research period. According to [3], the number of seeds per grape berry varied from 1.27 to 2.07, and these results were obtained in open pollination conditions. Certain research shows that there were no significant differences in fruit setting and seed number per berry between self-fertilization and cross-fertilization [22]. The pollenizer’s influence on the mechanical composition of the indigenous variety ‘Grk’, depending on the pollenizers that were included in the experiment (‘Plavac mali’, ‘Prošip bijeli’, and ‘Chardonnay’), were monitored by [45]. The highest value of grape stalk mass and the largest average number of seeds per 100 grape berries were achieved (133.81 g) when ‘Plavac mali’ was used as the pollenizer. The highest number of seeds in grape berries in the variety ‘Grk’ was obtained when ‘Plavac mali’ (49.17) and ‘Prošip bijeli’ (45.17) were used as pollenizers. The difference between these two pollenizers was not significant; however, the Chardonnay variety had a significantly different number of grape berries compared to the first two pollenizers (17.27). According to the same authors, during the berry analysis, the average number of seeds per berry in the variety ‘Grk’ was obtained, and it showed that no significant difference was found between the ‘Chardonnay’—1.07, ‘Prošip bijeli’—1.18, and ‘Plavac mali’—1.34 pollenizers.

The weight of one seed within the research period was slightly higher in varieties with larger berries, which was somewhat expected. A significant deviation in seed mass was registered during the open pollination in 2016. Earlier research by [13] shows that the average seed mass in the ‘Blatina’ variety in the vineyard in the 2008–2010 time period was 0.048 g and that it was in line with the results of this study. The use of pollen in the table varieties’ pollination significantly affected the seed mass, which was the highest in open pollination, then manual (controlled) pollination, and the lowest in self-pollination [2]. The significant influence of pollenizers on the berry characteristics (mass, length, and width) was stated by author [23] in his research.

## 4. Materials and Methods

### 4.1. Climatic Conditions

**Figure 11:**
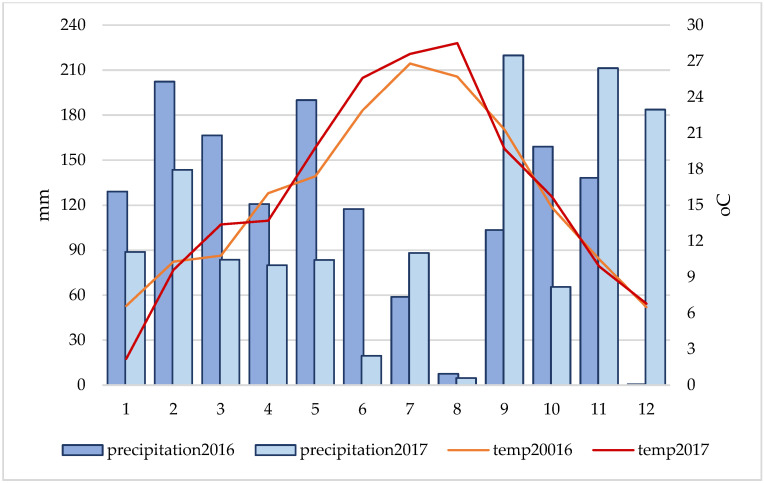
Climatic indicators (precipitation and temperature) during the research period (2016–2017) for the Mostar city area (meteorological station closest to the location of the vineyard where the research was conducted).

The experimental part of the research was realized during the years of 2016 and 2017 in the company ‘Agroherc d.o.o.’ Čapljina vineyard at the locality Višići-Čapljina, Bosnia and Herzegovina (43°03′55.4 N, 17°42′34.6 E, and 10 m above sea level). During 2017, a lower amount of precipitation was recorded in the period of flowering and fruit setting (Figure 11), compared to 2016, with slightly higher air temperatures in the same period [46].

The region is characterized by large amounts of precipitation during the year with an irregular schedule, while very high temperatures are registered during the summer months.

### 4.2. Plant Material

The vineyard was raised in 2008, and it occupies a total area of 67 ha. The main variety in the vineyard is ‘Blatina’, and there is also a large number of other economically important varieties. All tested varieties were grafted on a ‘Kober 5BB’ rootstock. The training system is Moser’s cordon, with the use of a short training system. Planting distance in the vineyard for all cultivars is 3.0 × 1.2 m, with a density of 2700 vines per hectare. A total of 90 individual grapevine inflorescence (flower cluster) on five vine plants of each variety was labeled before the beginning of flowering (during the entire research period, 180 individual grapevine inflorescence). The study was conducted on three replicates that consisted of five inflorescence clusters, with about 400–560 flower buds per cluster. The average number of flowers in the inflorescence was obtained by counting ten inflorescences for each combination. Analysis of the number of flowers in the inflorescence was performed in the BBCH 57 phase [47].

### 4.3. Pollination Treatments

In mid-May, about five to seven days before the anthesis, clusters of the ‘Blatina’ variety (75 of them) were enclosed within cheesecloth frames to avoid unwanted cross-pollination that can occur as well as the negative impact of weather conditions. For the open pollination treatment, flowers of 15 similar clusters were allowed to bloom and pollinate spontaneously according to the weather conditions. All tested cultivars, except ‘Cardinal’, were used as pollinators in open pollination. Each of these clusters was specifically marked at the beginning of the pollination period. Inflorescences marking (later grape clusters) was done by placing plastic labels with written numbers. The markings enabled the monitoring of selected clusters during the entire vegetation until the harvest phenophase. Manual pollination (cross-pollination) was performed during the bloom stage (BBCH 65 phase). The following varieties were used as pollenizers: ‘Trnjak’, ‘Alicante Bouschet’, ‘Žilavka’, ‘Vranac’, and ‘Cardinal’; these were grown in the same vineyard as the Blatina variety. The pollen of each pollenizer variety was collected by shaking the inflorescences onto petri dishes when most of the flowers were in full bloom. In the case of earlier flowering (‘Cardinal’ variety), the pollen was collected, sieved, and stored in glass bottles in a desiccator (with anhydrous CaSO_4_) at a refrigerator temperature of 4 °C. Pollen of other varieties was stored in the same way until the moment of pollination. The pollen germination test was performed in vitro using the “hanging drop” technique with a 20% sucrose solution [25,42]. Cross-pollination was carried out using a small pump (enema-rubber pump) containing the pollenizer pollen. As soon as the cluster reached receptivity, the pollination was implemented by blowing the pollen of each individual pollenizer variety, via pump, into the cheesecloth frames in the early morning (in the 2-h period between 8 and 10 a.m.). This procedure was repeated for two consecutive days to ensure that all the sequentially opening flowers in the inflorescence were pollinated [3]. An individual pump was used for each crossing combination in order to avoid pollen contamination. During both years of research, the same pollen application procedure was conducted. Seven days after fruit setting, the cheesecloths on self-pollination clusters were removed to expose the developing grapes to the sun throughout the development and maturation stages. The initial fruit set analysis was performed in the germinated phase (BBCH 71 phase).

### 4.4. Fruit Quality Characteristics

The harvest was performed at the time of optimal maturity for the ‘Blatina’ variety and when the TSS (Total Soluble Solid) content was between 18 and 22 Brix (BBCH 89 phase). The grape clusters were stored in portable containers and transported to the Laboratory for Ampelography and Winemaking of the Faculty of Agriculture, University of Banja Luka, for measurements and analysis. Individual clusters and mature grape berries of each treatment were collected, counted, and weighed. Fifteen representative clusters from each treatment were selected. The number of grape berries per cluster was recorded at maturity. For each treatment, the mass of grapes in the cluster and the mass of an individual grape berry were measured with a digital scale (KERN 440). Afterwards, the seeds from each mature grape berry were extracted and washed to remove pulp. The seeds were then submerged into water to separate viable and unviable seeds. The seeds that floated were washed and counted as viable, and, after that, they were cleaned and dried. The number of seeds in one grape berry for each of the treatments was counted. For the seed mass analysis, 10 viable seeds per combination were randomly selected, and their mass was measured on a digital scale.

### 4.5. Statistical Analysis

The statistical analysis included descriptive analysis (means and standard deviations for each pollinizer in each year of research as a whole). Levene’s test of homogeneity of variances was used. The significance of main and interaction effects was analyzed using two-way variance analysis (factorial ANOVA) (6 × 2). The factors included pollenizers (Open pollination, ‘Trnjak’, ‘Alicante Bouschet’, ‘Žilavka’, ‘Vranac’, ‘Cardinal’) and the year of research (2016, 2017). Tukey’s HSD post hoc test was used to test the differences between pollenizer pairs. A criterion α level of *p* ≤ 0.05 was used to determine statistical significance. The data was processed using the STATISTICA 10.0 for Windows (StatSoft, Inc., Tulsa, OK, USA).

## 5. Conclusions

The influence of different pollenizers on the variety ‘Blatina’ grape and seed characteristics was examined during a two-year period (2016/2017). Varieties ‘Trnjak’, ‘Cardinal’, ‘Alicante Bouschet’, ‘Žilavka’, and ‘Vranac’ were used as pollenizers. Varieties ‘Trnjak’, ‘Žilavka’, and ‘Vranac’ are indigenous varieties, and they have been grown for a long period of time in this area. This research shows that weather conditions can have a significant influence on fertilization and cluster and seed characteristics. Heavy precipitation and lower average air temperatures during the flowering phase and fertilization can have a negative effect on fruit set and, later, the cluster characteristics. Study of the influence of different pollenizers on the grape cluster and grape seed characteristics in the ‘Blatina’ variety during fertilization shows that significant differences exist between them. The best grape cluster and seed characteristics are achieved in open pollination conditions compared to conditions where other varieties are used as pollenizers. Open pollination has an increase in grape cluster mass by an average of 55.6% to 148.5% and an increase in grape berries mass in cluster by 18.9% to 156.3% compared to other analyzed pollenizers. The ‘Vranac’ and ‘Žilavka’ varieties had a more significant influence on the observed parameters compared to other pollenizer varieties in controlled pollination conditions. The increase in the average cluster mass by pollination with these varieties was, on average, 24.5% to 59.3% higher compared to other pollenizers. This increase is especially significant, keeping in mind that variety ‘Blatina’ has a lower fruit set percentage in the absence of a suitable pollenizer. Pollination with the indigenous ‘Trnjak’ variety also had a fairly stable effect, with the lowest oscillations during the research period. The use of the variety ‘Trnjak’ as a pollenizer for the variety ‘Blatina’ can be somewhat justified if the producers decide to grow indigenous grape varieties. The use of a larger number of varieties for the ‘Blatina’ variety pollination, although it provides safer production, is not justified due to vineyard production organization and the application of regular agro- and ampelotechnical measures.

## Figures and Tables

**Table 1:** Test of mutual multivariate dependence of pollenizer effects and year on basic grape and cluster characteristics.

	Wilks Value	F	Effect-df	Error-df	*p*
Variety	0.491	2.53	30	390	0.000 *
Year	0.741	5.64	6	97	0.000 *
Variety × Year	0.639	1.54	30	390	0.037 *

Legend: F—value of the Wilks test for analyzing the significance of difference between the centroid of groups; Effect df, Error df—degrees of freedom; *p*—significance coefficient. * statistically significant difference of observed parameters.

**Table 2:** Univariate results of the dependence of pollenizer effects and year of research on the basic cluster and grape characteristics.

Variable	Pollenizer		Year		Pollenizer × Year	
	F (5;102)	*p*	F (1;102)	*p*	F (5;102)	*p*
Cluster mass (g)	10.13	0.000 *	9.92	0.002 *	1.52	0.190
Berries mass (g)	9.06	0.000 *	11.62	0.001 *	1.49	0.201
Berries number	9.14	0.000 *	14.20	0.000 *	2.52	0.034 *
Berry mass (g)	1.80	0.119	3.00	0.086	2.99	0.015 *
Seed mass (g)	1.07	0.384	2.17	0.144	1.27	0.281
Seed number	2.02	0.082	6.10	0.015 *	3.17	0.011 *

Legenda: F—value of the Wilks test for analyzing the significance of difference between arithmetic means of groups; *p*—significance coefficient; *—statistically significant differences.

## Data Availability

The data presented in this study are available on request from the corresponding author. The data are not publicly available because they are the part of the research that has continued.

## References

[1] Costantini L., Moreno-Sanz P., Nwafor C.C., Lorenzi S., Marrano A., Cristofolini F., Gottardini E., Raimondi S., Ruffa P., Gribaudo I. (2021). Somatic variants for seed and fruit set in grapevine. BMC Plant Biol..

[2] Sabir A., Kilinc S., Sabir F. (2020). Qualitative and quantitative response of early ripening table grape cultivars (*Vitisvinifera* L.) to pollination treatments under controlled growing condition. Erwerbs-Obstbau.

[3] Sabir A. (2014). Xenia and metaxenia in grapes: Differences in berry and seed characteristics of maternal grape cv. ‘Narince’ (*Vitisvinifera* L.) as influenced by different pollen sources. Plant Biol..

[4] Kevan P., Blades D., Posluszny A., Ambrose J. (1988). Pollen dimorphisam and dioecy in *Vitisaestivalis*. Vitis.

[5] Silva P.R., Bione N., Silva N., Paglaini M.S. (2001). Meiotic behavior of the Brazilian table grape cultivar rubi (*Vitisvinifera* L.) with a high proportion of seed less berries. Vitis.

[6] Caporali E., Spada A., Marziani G., Failla O., Scienza A. (2003). The arrest of development of abortive reproductive organs in the unisexual flower of *Vitisvinifera* ssp. *silvestris*. Sex. Plant Reprod..

[7] Slimane-Harbi M.B., Chabbouh N., Snoussi H., Bessis R., Gazzah M. (2004). Etude du germoplasme de vignesautochtones de Tunisie. Precisions sur I’origine du millerandage du “Razzegui”. Bull. O.I.V..

[8] Abreu I., Costa I., Oliveria M., Cunha M., De Castro R. (2006). Ultrastructure and germation of *Vitisvinifera* L. cv Loureiropollen. Protoplasma.

[9] Gallardo A., Ocete R., Angeles Lopez M., Lara M., Rivera D. (2009). Assessment of pollen dimorphisim in populations of *Vitisvinifera* L. subsp. sylvestris (Gmelin) Hegi in Spain. Vitis.

[10] Najmaddin C., Khatijah H., Maideen H. (2011). Comparative study on the anatomy and palynology of the three variety of *Vitisviniferavarity* (family *Vitaceae*). Afr. J. Biotechnol..

[11] Sabir A., Sabir F., Kara Z., Yasin G., Mohammed O.J.M., Jawshle A.I.M., Kus A.D. (2019). Berry set and Quality Repsonse of Soilless grown “Prima” grapes to foliar inflorescence pulverization of various substances under glasshouse condition. Erwerbs-Obstbau.

[12] Jovanović-Cvetković T., Mićić N., Đurić G., Cvetković M. (2016). Pollen morphology and germination of indigenous grapevine cultivars Žilavka and Blatina (*Vitisvinifera* L.). AgroLife Sci. J..

[13] Jovanović-Cvetković T., Mijatović D., Grbić R. (2016). Effect of climatic parameters on uvological characteristics of variety “Blatina”. Ann. Univ. Craiova Agric. Montanology Cadastre Ser..

[14] Sabir A., Kucukbasmaci H. (2020). Agronomic response of ‘Michele palieri’ (*Vitisvinifera* L.) table grape to intraspecific diploid and interspecific tetraploid pollinizers. Sci. Hortic..

[15] Denney J.O. (1992). Xenia includes metaxenia. Hortic. Sci..

[16] Milutinović M., Nikolić D., Fotirić M., Rakonjac V. (2000). The relation between pollen functional ability and fruit set in grapevine (Vitis sp.). Genetika.

[17] Kimura P.H., Ükamoto G., Hirano K. (1988). Artificial pollination in *Vitiscoignetiae* Pulliat. Vitis.

[18] Kelen M., Demitras I. (2003). Fertilization biology of some grape varieties (*Vitis vinifera* L.). Pak. J. Biol. Sci..

[19] Mullins L. (1992). Biology of the Grapevine.

[20] Sharafi Y., Bahmani A. (2011). Pollen germination, tube growth and longevity in some cultivars of *Vitisvinifera L.*. Afr. J. Microbiol. Res..

[21] Burcak I. (2021). Pollen characteristics of some grape cultivars (Vitisvinifera L.). Int. J. Agric. Environ. Food. Sci..

[22] Sabır A. (2011). Influences of self-and cross-pollinations on berry set, seed characteristics and germination progress of grape (*Vitisvinifera* cv. Italia). Int. J. Agric. Biol..

[23] Sahin G. (2016). Effects of different pollinizers on berry set with berry and seed features in Alphonse Lavallee (*V.vinifera* L.). Master’s Thesis.

[24] García-Breijo F., Armiñana J.R., Garmendia A., Cebrián N., Beltrán R., Merle H. (2020). In Vivo Pollen Tube Growth and Evidence of Self-Pollination and PrefloralAnthesis in cv. Macabeo (*Vitisvinifera* L.). Agriculture.

[25] Kara Z., Sabir A., Doğan O., Khaleel A.J.K. (2017). Fertilization Biology of Ancient Grapevine Variety ‘Ekşi Kara’ (*Vitis vinifera* L.). Selcuk J. Agr. Food Sci..

[26] Toth M., Gaal M., Bodor P. (2005). Metaxenic pollen effect of scab resistant apple cultivars on the fruit of apple. Int. J. Hortic. Sci..

[27] Bodor P., Gaal M., Toth M. (2008). Metaxenia in apples cv. ‘Rewena’, ‘Relinda’, ‘Baujade’ as influenced by scab resistant pollinizers. Int. J. Hortic. Sci..

[28] Millitaru M., Butac M., Sumedrea D., Chitu E. (2015). Effect of metaxenia on the fruit quality of scab resistant apple varieties. Agric. Agric. Sci. Procedia.

[29] Tufts W.P., Hansen C.T. (1993). Xenia and metaxeniain the Bartlett pear. Proc. Am. Soc. Hort. Sci..

[30] Radičević S., Cerović R. (2015). New sour cherry (Prunuscerasus L.) cultivars developed at Fruit Research Institute in Cacak. J. Pomol..

[31] Ansari M., Davarynejad G.H., Tornyai J., Nyeki J., Szabo Z., Soltesz M. (2010). Effects of self- and crosspollination on fruit set and fruit quality cherry cultivars. Int. J. Hortic. Sci..

[32] Beljo J., Mandić A., Jovanović-Cvetković T., Dodig R., Gašpar M., Ivanković M., Jakirović A., Lasić V., Leko M., Nikić A. (2017). Atlas of Viticulture and Wine of Bosnia and Herzegovina.

[33] Štajner N., Tomić L., Ivanišević D., Korać N., Jovanović-Cvetković T., Beleski K., Angelova E., Maraš V., Javornik B. (2014). Microsatellite inferred genetic diversity and structure of Western Balkan grapevines (*Vitisvinifera* L.). Tree Genet. Genomes.

[34] Dallakyan M., Esoyan S., Gasparyan B., Smith A., Hovhannisyan N. (2020). Genetic diversity and traditional uses of aboriginal grape (*Vitisvinifera* L.) varieties from the main viticultural regions of Armenia. Genet. Resour. Crop. Evol..

[35] Blesić M. (2001). Stabilnost bojenih materija i kvalitet vina u zavisnosti od uslova vođjenja maceracije kljuka Blatine. Ph.D. Thesis.

[36] ŽuljMihaljević M., Šimon S., Pejić I., Carka F., Sevo R., Kojić A., Gaši F., Tomić L., Jovanović-Cvetković T., Maletić E. (2013). Molecular characterization of old local grapevine varieties from South East European countries. Vitis.

[37] Beleski K., Nedelkovski D. (2015). Identification and classification of grapevine cultivars (*Vitisvinifera* L.) from the Balkan subgroup by phyllometric descriptors. Vitis.

[38] Mandić A., Žulj Mihaljević M., Leko M., Primorac J., Beljo J. (2019). Synonymys and homonymus in Herzegovinian and Dalmatian grapevine cultivars. Acta Hortic..

[39] Bouard J. (1971). Tissusetorganes de la vigne. Traite d’Ampelologie: Sciences e Techniques de la Vigne.

[40] Mićić N., Đurić G., Jovanović-Cvetković T., Cvetković M. (2018). Pollen functional ability in two indigenous grapevine cultivars in Bosnia and Herzegovina. Eur. J. Hortic. Sci..

[41] Jovanović-Cvetković J. (2013). Anatomsko-morfološka i citohistološka evaluacija reproduktivnih organa autohtonih sorti vinove loze BiH.

[42] Kurtović M., Mijatović D., Blesić M., Tarailo R. (1989). Klijavost polena vinskih i stonih sorti vinove loze (Vitis vinifera L.). Poljopr. Pregl..

[43] Weaver R.J., McCune S.B. (1960). Further studies with gibberellin on Vitis vinifera grapes. Bot. Gaz..

[44] Tarailo R., Milošević G., Živković J., Ranković V., Stanković S. (1998). Perspektivni genotipovi vinove loze za stono grožđe i vino stvoreni u Nišu. Poljoprivreda.

[45] Stupić D. (2016). Reducirana oplodnja cv. Grk (*Vitis vinifera* L.) i njen utjecaj na kvalitetu grožđa i vina. Ph.D. Thesis.

[46] Federalni Hidrometeorološki Zavod BiH. http://www.fhmzbih.gov.ba.

[47] Growth Stages of Mono-and Dicotyledonous Plants. https://www.politicheagricole.it/flex/AppData/WebLive/Agrometeo/MIEPFY800/BBCHengl2001.pdf.

